# Natural Killer Cell Mediated Antibody-Dependent Cellular Cytotoxicity in Tumor Immunotherapy with Therapeutic Antibodies

**DOI:** 10.3389/fimmu.2013.00076

**Published:** 2013-03-27

**Authors:** Ursula J. E. Seidel, Patrick Schlegel, Peter Lang

**Affiliations:** ^1^Department of General Paediatrics, Oncology/Haematology, University Children’s Hospital TübingenTübingen, Germany

**Keywords:** natural killer cells, ADCC, tumor immunotherapy, therapeutic antibodies, allogeneic stem cell transplantation

## Abstract

In the last decade several therapeutic antibodies have been Federal Drug Administration (FDA) and European Medicines Agency (EMEA) approved. Although their mechanisms of action *in vivo* is not fully elucidated, antibody-dependent cellular cytotoxicity (ADCC) mediated by natural killer (NK) cells is presumed to be a key effector function. A substantial role of ADCC has been demonstrated *in vitro* and in mouse tumor models. However, a direct *in vivo* effect of ADCC in tumor reactivity in humans remains to be shown. Several studies revealed a predictive value of FcγRIIIa-V158F polymorphism in monoclonal antibody treatment, indicating a potential effect of ADCC on outcome for certain indications. Furthermore, the use of therapeutic antibodies after allogeneic hematopoietic stem cell transplantation is an interesting option. Studying the role of the FcγRIIIa-V158F polymorphism and the influence of Killer-cell Immunoglobuline-like Receptor (KIR) receptor ligand incompatibility on ADCC in this approach may contribute to future transplantation strategies. Despite the success of approved second-generation antibodies in the treatment of several malignancies, efforts are made to further augment ADCC *in vivo* by antibody engineering. Here, we review currently used therapeutic antibodies for which ADCC has been suggested as effector function.

## Introduction

Naturally cytotoxic cells against tumor cells were first described in humans and mice in the 1970s of the last century (Rosenberg et al., 1972; Oldham and Herberman, 1973; Herberman et al., 1975a,b; Kiessling et al., 1975a,b). Initially regarded as artifacts, these cells were recognized eventually as a novel lymphocyte population and named natural killer (NK) cells after their natural occurrence and spontaneous capacity to kill lymphomas and leukemic cells in non-immunized animals (Kiessling et al., 1975a,b). Nowadays, NK cells are recognized as a subset of cytotoxic innate lymphoid cells (ILCs) which are able to directly kill virus-infected cells and tumor cells and participate in shaping the adaptive immunity by secretion of cytokines (e.g., IFN-γ) (Vivier et al., 2011). The role of NK cells in human cancer is highlighted by a study linking low peripheral blood NK cell activity with increased cancer risk (Imai et al., 2000). Furthermore, an association of NK cell infiltration into the tumor site with better disease prognosis has been shown for several malignancies (Carrega et al., 2008; Halama et al., 2011; Platonova et al., 2011; Eckl et al., 2012).

**Figure 1:**
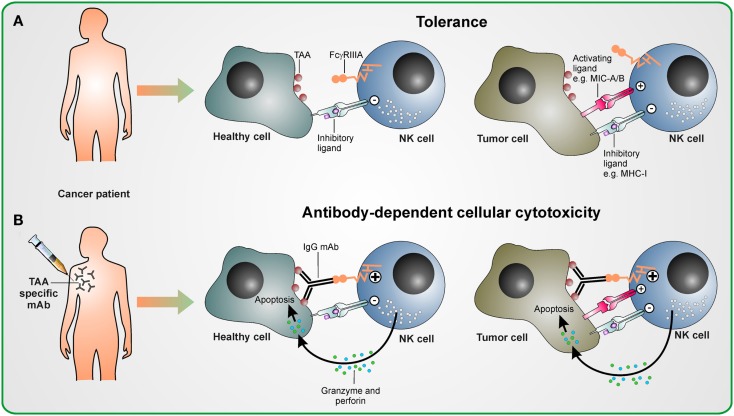
**Antibody-dependent cellular cytotoxicity in therapeutic antibody treatment**. **(A)** Without antibody therapy, NK cells are tolerant to healthy cells and tumor cells, if the strength of activating signal they receive upon encountering activating ligands on these malignant cells does not overcome the inhibitory signaling delivered by inhibitory ligands as, e.g., MHC class I molecules. **(B)** Upon treatment with tumor antigen-associated (TAA) specifc antibody, the activating stimulus from FcγRIIIa induced by antibodies cross-linking NK cells with TAA-expressing healthy and malignant cells overcomes inhibitory signals. This leads to the activation of NK cells and ADCC is mediated by releasing cytotoxic granules containing perforin and granzyme.

Human NK cells are defined by the phenotype CD3^−^ CD56^+^; additionally they are CD19 and CD14 negative. The only marker that is specific for NK cells is NKp46. NK cells comprise 5–15% of all circulating lymphocytes (Lanier et al., 1986; Walzer et al., 2007). They are commonly divided into two major subpopulations, CD56^dim^ CD16^+^ and CD56^bright^ CD16^−^, with each of those possessing distinct effector functions. The CD56^dim^ CD16^+^ subset comprises 90% of all peripheral blood NK cells and mediates an early response via direct cellular cytotoxicity induced by perforin and granzyme, FasL, and TRAIL interactions as well as cytokine production (De Maria et al., 2011). One major characteristic of NK cells is their constant state of readiness to respond immediately. In contrast to T cells, NK cells constitutively express perforin. This facilitates the instant polarized delivery of apoptosis-inducing granzymes after formation of a lytic synapse between the NK cell and a target cell (Shresta et al., 1995). The CD56^bright^ CD16^−^ subset mediates a late but sustained effector function via potent pro-inflammatory cytokine and chemokine release of mainly IFN-γ, but is poorly cytotoxic (De Maria et al., 2011). NK cell activation and cytotoxicity is controlled by a complex balance between activating receptors, inhibitory receptors and co-receptors (Lanier, 2003; Leung, 2011). Positive and negative downstream signals of these receptors are integrated and decisive for NK cell activation (Figure 1). Hence, absence of inhibitory signals on target cells together with engagement of activating receptors as NKG2D, DNAM-1, and 2B4 as well as the natural cytotoxicity receptors (NCRs), including NKp46, NKp44, and NKp30 mediate triggering of resting NK cells (Moretta et al., 2001; Bryceson and Long, 2008; Lanier, 2008). Whereas for NKG2D, DNAM-1, and 2B4 multiple ligands are known (Bottino et al., 2005), despite their involvement in tumor cell lysis the NCR ligands have remained rather elusive (Moretta et al., 2006). Nevertheless, NKp30 has been shown to recognize a tumor cell ligand of the B7 family, B7-H6 (Brandt et al., 2009) and NKp44 is suggested to recognize proliferating cell nuclear antigen (PCNA), which surprisingly triggers inhibition of NK cells (Rosental et al., 2011). However, several of those receptors need to be triggered by target cells for activating downstream signals to prevail over inhibitory signals and NK cells to be activated and to mediate target cell lysis (Moretta et al., 2001; Bryceson et al., 2006).

## Antibody-Dependent Cellular Cytotoxicity

Most hematopoietic cells, except most T cell subsets, express Fcγ receptors (FcγRs) (Nimmerjahn and Ravetch, 2008). There are three types of FcγRs which recognize the Fc part of IgG antibody subclasses with different affinities. The activating FcγRI (CD64) binds to human IgG1 and IgG3 with high affinity, is expressed on macrophages and neutrophils, and mediates phagocytosis of target cells. The FcγRII (CD32) class comprises activating low affinity FcγRIIa (which binds human IgG1, IgG2, and IgG3) and inhibitory FcγRIIb (which recognizes human IgG1 and IgG3 with low affinity) and may attenuate signaling from activating receptors as FcγRI if engaged in phagocytes. FcγRIIIb is a protein expressed by neutrophils and may play a role in neutrophil activation. Activating low affinity FcγRIIIa (type III receptor for IgG; CD16) mediates antibody-dependent cellular cytotoxicity (ADCC) and is highly expressed on the cytotoxic CD56^dim^ CD16^+^ NK cell subset as well as on other hematopoietic cells. Nevertheless, NK cells are thought to be the key mediators of ADCC, since only NK cells do not co-express the inhibitory FcγRIIb. In contrast, all other FcγR-expressing cells are tightly controlled by the balance between activating and inhibitory FcγRs. Efficient FcγRIIIa signaling depends on high avidity for specific binding to antibodies which ensures that NK cells are activated when antibodies have bound to a multimeric or multivalent cognate antigen only (Banks et al., 2002). These antibodies of the subclasses IgG1 and IgG3 binding to FcγRIIIa induce a potent activating signal which overcomes inhibitory signals and results in both cytotoxicity and a cytokine response (Chan et al., 2012) (Figure 1). Co-engagement of other activating receptors has a synergistic effect and may enhance NK cell activation (Bryceson et al., 2006).

Several mouse model studies postulated the impact of activating FcγRs on anti-tumor effects in antibody therapy indicating that ADCC has a substantial effect on tumor rejection. A study using a xenograft breast carcinoma model has shown that an antibody engineered to prevent Fc binding to FcγRIIIa and specific to Her2/neu [Human Epidermal Growth Factor Receptor (EGFR) 2, CD340] which is overexpressed in certain aggressive types of breast cancer, was, in contrary to its murine parental antibody targeting Her2/neu, unable to arrest tumor growth *in vivo* (Clynes et al., 2000). Additionally, this study in mice showed in a murine lymphoma model that treatment with CD20-specific antibody rituximab was capable of preventing tumor growth in FcR wild-type mice whereas FcR knock-out mice failed to mediate antibody-dependent tumor protection. Another study demonstrated ADCC in mice *in vivo* by showing formation of ADCC synapses in immunocompetent mice bearing a murine breast tumor treated with an antibody specific to Tn, a glycopeptidic antigen which is expressed by breast cancer and a variety of other epithelial tumors in mice and humans (Hubert et al., 2011). Additionally, tumor rejection was abolished in mice deficient for FcγRs – emphasizing the potential role of ADCC (Hubert et al., 2011).

Another study investigated the relative contributions of complement-dependent cytotoxicity (CDC) and ADCC in a murine GD2-expressing metastatic lymphoma model treated with an antibody specific to the disialoganglioside GD2 utilizing wild-type, complement-deficient, complement-receptor-deficient, and FcγRI/III-deficient mice. Outcome after treatment with the ADCC and CDC mediating GD2 antibody was unaffected in mice incapable for CDC but was almost completely abrogated in FcγRI/III-deficient mice that were disqualified for ADCC (Imai et al., 2005). These results further highlight the key role of ADCC in anti-tumor effects in mice *in vivo*. In men it has been shown, that breast cancer patients who responded with partial or complete remission to Her2/neu-specific antibody trastuzumab have a higher capacity to mediate ADCC *in vitro* than patients which failed to respond to antibody therapy (Musolino et al., 2008).

These studies indicate that, at least in murine models, ADCC is a considerable component of the *in vivo* activity of therapeutic antibodies against tumors. However, studies with therapeutic antibodies in humans which demonstrate the direct influence of ADCC, as, e.g., ADCC as the single effector mechanism, remain to be conducted. Therefore, the direct *in vivo* effect of ADCC in tumor reactivity in humans remains to be shown.

## Influence of FcγRIIIa Polymorphism and Killer-Cell Immunoglobuline-Like Receptor (KIR) Receptor Ligand Incompatibility on ADCC in Patients

In the past 15 years, 12 therapeutic antibodies have reached FDA approval for hematological malignancies as well as solid tumors (Scott et al., 2012). Their mechanisms of action include direct anti-tumor effects as induction of apoptosis, blocking receptor signaling or acting as an agonist, delivery of a cytotoxic agent, immune-mediated effects as CDC and ADCC as well as effects on the tumor microenvironment. At least five of these antibodies mediate efficient ADCC and a large number of new constructs are currently under investigation in early and late phase clinical trials (Table 1). In this review we will use anti-CD20-specific monoclonal antibody (mAb) rituximab, CD52-specific mAb alemtuzumab, Her2/neu-specific mAb trastuzumab, EGFR-specific mAb cetuximab, and anti-GD2 antibodies to discuss the influence of ADCC in treatment with therapeutic antibodies.

**Table 1:** **ADCC-mediating therapeutic antibodies currently FDA approved for cancer therapy**.

Antibody	Antigen	Cancer indication	Mechanisms of action
Rituximab	CD20	CD20^+^ B cell NHL, CD20^+^ follicular NHL, CLL	ADCC, CDC, direct induction of apoptosis
Ofatumumab	CD20	CLL	ADCC, CDC
Trastuzumab	Her2/neu	Breast cancer	ADCC, abrogation of tumor cell signaling
Cetuximab	EGFR	colorectal cancer, SCCHN	ADCC, abrogation of tumor cell signaling
Alemtuzumab*	CD52	CLL	ADCC, CDC, direct induction of apoptosis

*NHL, non-Hodgkin’s lymphoma; CLL, chronic lymphocytic leukemia; ADCC, antibody-dependent cellular cytotoxicity; CDC, complement-dependent cytotoxicity; SCCHN, squamous cell carcinoma of the head and neck*.

*Withdrawn from the market in August, 2012.

The CD16 encoding gene FCGR3A bears a single nucleotide polymorphism (SNP) at nucleotide 526 [thymidine (T) → guanine (G)] resulting in an amino acid (aa) exchange at aa position 158 of phenylalanine (F) to valine (V). It has been demonstrated that human IgG1 binds more efficiently to NK cells expressing the FcγRIIIa-158V allotype than to the FcγRIIIa-158F allotype (Koene et al., 1997; Wu et al., 1997). *In vitro* studies have shown that the increased binding is caused by a significantly higher affinity of IgG antibodies to FcγRIIIa-158V receptors, whereas expression levels of FcγRIIIa on NK cells are not influenced by the FcγRIIIa polymorphism (Dall’Ozzo et al., 2004; Congy-Jolivet et al., 2008). Furthermore, the adequate rituximab concentration exciting 50% lysis of a CD20^+^ tumor cell line has been shown to be significantly lower in FcγRIIIa-158V/V donors compared to FcγRIIIa-158F/F donors (Dall’Ozzo et al., 2004).

Several clinical studies investigating antibodies which utilize an ADCC-mediating therapeutic antibody suggest that patients with the FcγRIIIa-158V allotype have a better clinical outcome. However, conflicting data have been published. First, we will discuss the chimeric IgG1 CD20-specific antibody rituximab which is approved for treatment of CD20^+^ B-cell non-Hodgkin’s lymphoma (NHL), CD20^+^ follicular NHL, and chronic lymphocytic leukemia (CLL). Rituximab is the most extensively studied antibody mediating ADCC as its key effector mechanism (Cheson and Leonard, 2008; Alduaij and Illidge, 2011). The first study by Cartron et al. (2002) suggested that the homozygous FcγRIIIa-158V/V genotype is beneficial in patients with follicular NHL regarding clinical and molecular responses to single agent rituximab. These results were confirmed in a larger follicular lymphoma patient cohort treated with rituximab as monotherapy and an association of the FcγRIIIa-158V/V genotype with progression-free survival could be shown (Weng and Levy, 2003). Another study analyzing the effect of the FcγRIIIa genotype on the outcome of patients to single agent rituximab promoted some of the above mentioned reports by suggesting a significant improvement in event-free survival in FcγRIIIa-158V/V patients (Ghielmini et al., 2005). Furthermore, it has been postulated that NK cells from lymphoma patients with the FcγRIIIa-158V allotype but not from patients that are homozygous for FcγRIIIa-158F/F were activated after rituximab application (Veeramani et al., 2011).

On the contrary, no influence of the FcγRIIIa-F158V polymorphism on the outcome of follicular lymphoma patients treated with risk-adapted chemotherapy and rituximab has been shown (Prochazka et al., 2011). Similarly, in relapsed or refractory CLL patients treated with chemotherapy and rituximab, no significant influence of the FcγRIIIa-F158V polymorphism was demonstrated (Dornan et al., 2010). Another study evaluated the impact of the FcγRIIIa-F158V polymorphism on the response to rituximab in combination with a chemotherapy regimen (rituximab with cyclophosphamide, hydroxydaunorubicin, oncovin, and predniso(lo)ne, R-CHOP) in diffuse large B cell lymphoma (DLBCL) patients. The FcγRIIIa-158V allotype was shown to be significantly correlated with a higher complete response rate compared with the FcγRIIIa-158F allotype (Kim et al., 2006). However, overall survival did not correlate with the FcγRIIIa-158V/V genotype and several other studies investigating the impact of the FcγRIIIa-F158V polymorphism in DLBCL found no association with the response to R-CHOP (Carlotti et al., 2007; Mitrovic et al., 2007; Varoczy et al., 2012). Interestingly, all studies conducted with rituximab as monotherapy show an impact of the FcγRIIIa-F158V polymorphism and therefore indicate an important influence of ADCC on clinical outcome. However, the picture is less clear when rituximab is combined with chemotherapy. Since combined chemotherapy may hamper ADCC due to impaired NK cell function the combination of rituximab with chemotherapy might mask the influence of the FcγRIIIa genotype and ADCC. Nevertheless, a combination of rituximab with chemotherapy generally improves clinical results.

The humanized IgG1 mAb alemtuzumab was approved for treatment of B-CLL and is directed against the glycoprotein CD52 (Hillmen et al., 2007). The therapeutic antibody was withdrawn from the market in 2012 in order to optimize the pending launch of the agent as a treatment for multiple sclerosis. The predominant mechanism of action of alemtuzumab *in vivo* has not been clearly defined but involvement of ADCC mediated by NK cells and neutrophils has been suggested by studies employing a human CD52 transgenic mouse model and a xenograft model (Hu et al., 2009; Siders et al., 2010). In addition, CDC and direct induction of apoptosis have been identified as other potential mechanisms of action in *in vitro* studies (Table 1) (Crowe et al., 1992; Mone et al., 2006). A study exploiting the predictive value of FcγRIIIa polymorphism in a small CLL patient cohort (*n* = 33) treated with alemtuzumab found no association of responsiveness to the mAb with a FcγRIIIa-F158V allotype (Lin et al., 2005). However, further studies with larger patient cohorts are required in order to determine the potential influence of FcγRIIIa-F158V polymorphism and ADCC in treatment with alemtuzumab.

The humanized IgG1 mAb trastuzumab is applicable in HER2/neu-positive breast cancer patients and mediates abrogation of tumor cell signaling and ADCC (Table 1) (Hudis, 2007). A study investigating breast cancer patients treated with trastuzumab and a chemotherapeutic agent postulated a higher response rate and longer progression-free survival in homozygous FcγRIIIa-158V/V patients compared to patients with the FcγRIIIa-158F allotype (Musolino et al., 2008). In contrast, another study suggested a correlation of the FcγRIIa-131H/H genotype with objective response and progression-free survival in HER2/neu-positive non-metastatic and metastatic breast cancer patients treated with chemotherapy and trastuzumab but no significant correlation was observed for the FcγRIIIa-F158V/V genotype (Tamura et al., 2011). Furthermore, another recent study postulated a lack of correlation between FcγRIIIa genotypes and trastuzumab efficacy in HER2/neu-positive non-metastatic breast cancer patients treated with a combined trastuzumab-chemotherapy (Hurvitz et al., 2012). Since no studies with trastuzumab as single agent have been published and besides ADCC abrogation of tumor cell signaling being a major effector mechanism of trastuzumab the influence of FcγRIIIa-F158V polymorphism and ADCC on clinical outcome is hard to substantiate.

The chimeric IgG1 therapeutic antibody cetuximab targets EGFR in colorectal cancer (CRC) and has like trastuzumab ADCC and abrogation of tumor cell signaling as the modes of action (Table 1) (Van Cutsem et al., 2009). A study suggested a significantly higher clinical benefit after treatment with cetuximab and chemotherapy in homozygous FcγRIIIa-158V/V and or FcγRIIa-131H/H patients. These results were independent from the GTPase KRas (KRAS) mutation status in metastatic CRC (mCRC) (Bibeau et al., 2009). Furthermore, a higher disease control rate in KRAS-mutated mCRC patients harboring the FcγRIIa-131H/H genotype and treated with combined cetuximab-chemotherapy has been shown (Rodriguez et al., 2012). It has been suggested that the predictive value of FcγRIIa-H131R polymorphism may result from linkage disequilibrium between FcγRIIIa-V158F and FcγRIIa-H131R and therefore nevertheless indicates an effect of ADCC (Lejeune et al., 2008). Similarly, the association of the FcγRIIIa-F158V polymorphism with progression-free survival has been suggested for single agent cetuximab treated mCRC patients (Zhang et al., 2007). However, in this study and in another study from Dahan et al. investigating combined cetuximab-chemotherapy treatment, the FcγRIIIa-158F allele rather than the FcγRIIIa-158V allele had a favorable influence on overall and progression-free survival (Zhang et al., 2007; Dahan et al., 2011). Beyond that, a recent study found no association of clinical outcome in a patient cohort of 107 mCRC patients treated with cetuximab and chemotherapy and FcγRIIIa-F158V and FcγRIIa-H131R polymorphisms (Park et al., 2012). These discrepancies related to the predictive value of the FcγRIIIa-F158V polymorphism on cetuximab treatment are difficult to explain and indicate a demand for further elaborated investigations in larger patient cohorts. Interestingly, mutated KRAS induces a ligand-independent activation of EGFR downstream signaling pathways. Hence, the key effector mechanism of cetuximab besides ADCC, namely abrogation of tumor signaling, is invalidated by KRAS mutations. Clinical benefit of cetuximab yet seen in these patients and the predictive value of FcγRIIIa-V158F genotype emphasizes the impact of ADCC.

Disialoganglioside GD2 is a sialic acid containing glycosphingolipid which is uniformly expressed in neuroblastoma and most melanomas but also to a variable degree in some other tumors (Navid et al., 2010). Due to its tumor-associated expression, several anti-GD2 antibodies such as the chimeric IgG1 antibody ch14.18 and its humanized counterpart hu14.18 as well as 3F8 have been developed and deployed in the clinic. The mechanisms of actions of these antibodies are ADCC and CDC. Several years ago, a phase I study with pediatric neuroblastoma patients treated with anti-GD2 antibody ch14.18 was conducted at our institute and elicited some complete and partial tumor responses in neuroblastoma patients (Handgretinger et al., 1995). Currently, we are conducting an ongoing phase I/II-trial for relapsed metastatic neuroblastoma with subsequent immunotherapy with ch14.18/Chinese hamster ovary (CHO) after HLA mismatched, haploidentical stem cell transplantation (SCT) at our institution. Preliminary results show effective ADCC and complement-mediated anti-tumor effects against neuroblastoma cells with donor-derived NK cells *in vitro* as well as significant anti-tumor effects *in vivo* (unpublished data). Due to yet small patient cohorts, the role of FcγRIIIa-F158V polymorphism in treatment with GD2 antibodies has not been studied up to now. However, there are a variety of further studies ongoing which investigate the *in vivo* efficacy of GD2 antibodies (Navid et al., 2010; Shusterman et al., 2010; Yu et al., 2010; Alderson and Sondel, 2011; Simon et al., 2011). Yu et al. demonstrated in a randomized trial a significantly better event-free survival for patients who received a combination of ch14.18, GM-CSF, and interleukin 2 than patients without ch14.18. Additionally, the third-generation antibody hu14.18K322A, which bears an aa substitution in the CH2 part, has been suggested to induce increased dose-dependent ADCC compared to ch14.18 and hu14.18, whereas dose-limiting CDC is intercepted by the Fc modification (Navid et al., 2010). In fact, results from studies conducted with this antibody *in vivo* will be interesting regarding anti-tumor effects caused by ADCC as single mode of action without involvement of any other effector mechanisms.

In adult acute myeloid leukemia (AML) and pediatric acute lymphoblastic leukemia (ALL) donor versus recipient NK cell alloreactivity is a key mechanism after HLA mismatched, haploidentical SCT, and has been reviewed elsewhere (Velardi et al., 2012). These “unlicensed” NK cells are characterized in the autologous setting by lacking self-KIRs and are thought to be beneficial in patients with neuroblastoma as well (Venstrom et al., 2009; Delgado et al., 2010). Interestingly, a recent study investigating the differential potential for ADCC of “licensed” and “unlicensed” NK cells in neuroblastoma, showed that “unlicensed” NK cells mediate ADCC most effectively against neuroblastoma cell lines under inflammatory conditions (Tarek et al., 2012). Based on these studies and our ongoing trial with HLA mismatched, haploidentical stem cell transplanted neuroblastoma patients treated with ch14.18/CHO we suggest that in the allogeneic, HLA mismatched SCT setting, KIR receptor ligand incompatibility may have synergistic effects with subsequent antibody therapy and propose further exploration of this hypothesis in larger clinical trials.

## Enhanced ADCC by Third-Generation Monoclonal Antibodies

Presuming that ADCC is a key mediator of anti-tumor effects *in vivo*, enhancing ADCC by engineering mAbs is expected to markedly improve clinical efficacy of therapeutic antibodies. Two main approaches of optimizing FcγRIIIa binding by enhancing the affinity of mAbs have been suggested and shown to induce 5- to 100-fold increased ADCC *in vitro* in recent years: molecular modifications in the Fc portion leading to aa substitutions (Shields et al., 2001; Lazar et al., 2006; Stavenhagen et al., 2007) and modifying Fc-linked glycosylation (Umana et al., 1999; Davies et al., 2001; Shinkawa et al., 2003).

The approach of optimizing the Fc portion of a therapeutic antibody via aa substitutions predominantly intends to enhance ADCC by increasing the affinity to activating FcγRIIIa and reducing the affinity to inhibitory FcγRIIb. A considerable number of these third-generation monoclonal antibodies are currently under early clinical investigation. These therapeutic antibodies target among others CD19, CD20, CD30, CD40, and FLT3 in hematological malignancies and all of them aim at achieving highly augmented ADCC *in vivo* (Awan et al., 2010; Foyil and Bartlett, 2010; Horton et al., 2010; Le Garff-Tavernier et al., 2011; Hofmann et al., 2012; Kellner et al., 2013). Furthermore, a CD19-specific Fc-optimized therapeutic antibody is currently evaluated in pediatric patients with refractory acute B-lineage leukemia after allogeneic SCT within the scope of a compassionate use program at our institution. In four out of six patients at very high risk of relapse, minimal residual disease could be significantly reduced or completely eradicated with a longest follow up of 15 months (unpublished data). Other attempts aim at reduction of CDC related toxicity as applied in GD2 targeting hu14.18K322A in neuroblastoma (Lazar et al., 2006; Sorkin et al., 2010) or enhancing affinity to low affinity allele FcγRIIIa-158F (Bowles et al., 2006; Nordstrom et al., 2011). Recently, a phase I trial investigating a Fc-engineered antibody targeting CD20 in follicular lymphoma patients has suggested encouraging results even in patients with the less favorable FcγRIIIa-F allotype (Forero-Torres et al., 2012).

Glyco-engineered therapeutic antibodies either bear oligosaccharides modified by bisecting GlcNAc, a β1,4-GlcnAc residue attached to a core β-mannose residue, or lack the core fucose of the Fc oligosaccharides and have been found to exhibit significantly higher ADCC than their unmodified counterparts (Lifely et al., 1995; Shinkawa et al., 2003). These modifications of oligosaccharides are mainly obtained by either transfecting CHO cell line with *N*-acetylglucosaminyltransferase III (GnTIII) or eliminating its intrinsic α-1,6-fucosyltransferase (FUT8) activity. Alternative expression systems such as Sf21 insect cells, resulting in fucosylated paucimannosidic *N*-linked glycosylation, have been suggested to elicit an enhanced ADCC and have been investigated with a chimeric CD19 antibody at our institution (Lang et al., 2004; Barbin et al., 2006). Several other glyco-engineered antibodies targeting a variety of different tumor associated antigens like CD19, CD20, EGFR, and GD2 are currently investigated in early clinical studies (Robak, 2009; Navid et al., 2010; Paz-Ares et al., 2011; Ward et al., 2011). All glyco-engineered antibodies in clinical trials have been reviewed in more detail elsewhere (Beck and Reichert, 2012). Recently, the C–C chemokine receptor 4 (CCR4) targeting glyco-engineered antibody mogamulizumab has been approved in Japan for use in patients with relapsed and refractory CCR4-positive adult T cell leukemia/lymphoma (ATL) (Beck and Reichert, 2012). Clinical superiority of these third-generation mAbs over clinically established therapeutic antibodies remains to be shown and may further portend the key role of ADCC for clinical use.

## Conclusion

The studies discussed here clearly demonstrate a substantial role of ADCC *in vitro* and in mouse tumor models. However, the direct *in vivo* effect of ADCC in tumor reactivity in humans remains to be shown since no therapeutic antibody with ADCC as single mechanism of action has been investigated in clinical trials to date. Conflicting results have been obtained about the role of FcγRIIIa-V158F polymorphism in mAb treatment. Several studies have shown a predictive value, which underlines the influence of ADCC *in vivo* and suggests that ADCC is one of the key mechanisms for clinical efficacy of therapeutic antibodies. On the other hand, some studies could not confirm these results. Further studies investigating the clinical relevance of FcγRIIIa-V158F polymorphism may be required for each therapeutic antibody and its indications. Although additional chemotherapy can mask the influence of the FcγRIIIa genotype and may hamper ADCC due to impaired NK cell function, a combination of both is able to improve clinical results. In particular, the use of therapeutic antibodies after hematopoietic SCT is an interesting treatment option, as the new donor-derived immune system usually faces minimal tumor burden. Further exploration of the role of the FcγRIIIa-V158F polymorphism and the influence of KIR-receptor-ligand incompatibility on ADCC in allogeneic hematopoietic SCT may contribute to future transplantation strategies in this setting. Furthermore, clinical superiority of ADCC improved third-generation mAbs over clinically established second-generation antibodies remains to be shown and may further portend the key role of ADCC in cancer therapy with monoclonal antibodies.

## Conflict of Interest Statement

The authors declare that the research was conducted in the absence of any commercial or financial relationships that could be construed as a potential conflict of interest.
